# Nitric oxide as a mediator of gastrointestinal mucosal injury?—Say it ain't so

**DOI:** 10.1155/S0962935195000640

**Published:** 1995-11

**Authors:** Paul Kubes, John L. Wallace

**Affiliations:** 1 Gastrointestinal and Immunology Research Groups Faculty of Medicine University of Calgary Alberta Calgary Canada

## Abstract

Nitric oxide has been suggested as a contributor to tissue injury in various experimental models of gastrointestinal inflammation. However, there is overwhelming evidence that nitric oxide is one of the most important mediators of mucosal defence, influencing such factors as mucus secretion, mucosal blood flow, ulcer repair and the activity of a variety of mucosal immunocytes. Nitric oxide has the capacity to down-regulate inflammatory responses in the gastrointestinal tract, to scavenge various free radical species and to protect the mucosa from injury induced by topical irritants. Moreover, questions can be raised regarding the evidence purported to support a role for nitric oxide in producing tissue injury. In this review, we provide an overview of the evidence supporting a role for nitric oxide in protecting the gastrointestinal tract from injury.


Mediators of Inflammation 4, 397–405 (1995)

### Nitric oxide as a mediator of gastrointestinal mucosal injury?—Say it ain't so

Paul Kubes and John L. Wallace[CA]

Gastrointestinal and Immunology Research Groups, Faculty of Medicine, University of Calgary, Calgary, Alberta, Canada

[CA]Corresponding Author

**Nitric oxide has been suggested as a contributor to tissue injury in various experimental models of gastrointestinal inflammation. However, there is overwhelming evidence that nitric oxide is one of the most important mediators of mucosal defence, influencing such factors as mucus secretion, mucosal blood flow, ulcer repair and the activity of a variety of mucosal immunocytes. Nitric oxide has the capacity to down-regulate inflammatory responses in the gastrointestinal tract, to scavenge various free radical species and to protect the mucosa from injury induced by topical irritants. Moreover, questions can be raised regarding the evidence purported to support a role for nitric oxide in producing tissue injury. In this review, we provide an overview of the evidence supporting a role for nitric oxide in protecting the gastrointestinal tract from injury.**

**Key words:** Inflammation, inflammatory bowel disease, mucosal defence, ulcer

### Introduction

It is now widely accepted that nitric oxide (NO) synthesized from L-arginine is the substance that accounts for the activity originally described by Furchgott and Zawadzki as 'endothelium-derived relaxing factor'.[1] Nitric oxide is produced by virtually all mammalian cells. In the intestine, nitric oxide is produced by three distinct enzyme systems, referred to as nitric oxide synthases (NOS). A neurally associated, constitutive form of NOS found in neurons of the enteric nervous system is referred to as NOS1 or ncNOS. An inducible form of the enzyme, referred to as NOS2 or iNOS, is found under appropriate circumstances in endothelial cells, the epithelium and in a number of immunocytes. This enzyme can be induced by exposure to various cytokines and endotoxin. NOS3 (or ecNOS) is a constitutively expressed enzyme found primarily in the endothelium lining blood vessels, but may also exist in intestinal epithelial cells and mast cells. One of the important distinguishing features between the constitutive and inducible forms of NOS is the calcium-dependency of the former but not the latter. For the most part, NOS1 and NOS3 can be regarded as the forms that are responsible for producing nitric oxide in a physiological context, whereas NOS2 produces nitric oxide in pathophysiological circumstances. However, this statement should not be construed as meaning nitric oxide derived from NOS2 only acts in a detrimental manner.

In recent years, a plethora of studies have implicated NO as a critical mediator of mucosal defence in the gastrointestinal tract. On the other hand, it has been suggested by many groups that nitric oxide contributes to mucosal injury in several human gastrointestinal disorders and in experimental animal models of gastrointestinal injury. Like oxygen, high concentrations of NO oxide are cytotoxic. While there are considerable data suggesting that NO production is greatly elevated in situations of mucosal inflammation, such as in inflammatory bowel disease, it has yet to be clearly established that NO plays a primarily damaging role in those circumstances, or that it ever accumulates to concentrations that could achieve such results.

In this review, we summarize some of the key evidence suggesting a role for nitric oxide in the maintenance of gastrointestinal mucosal integrity, and we challenge the hypothesis that nitric oxide, when present in 'large' amounts, necessarily results in mucosal injury and dysfunction.

### Nitric oxide and mucosal defence

The mucosa of the gastrointestinal tract is continuously exposed to potentially damaging substances, such as acid, digestive enzymes, bacterial toxins and bile. The ability of the mucosa of the gastrointestinal tract to resist injury by these sub-

© 1995 Rapid Science Publishers

**Table 1.** Effects of inhibition of nitric oxide synthase or administration of nitric oxide donors in models of gastrointestinal injury

| Model of injury | Inhibition of nitric oxide synthase | Exogenous nitric oxide administration |
|---|---|---|
| Hydrochloric acid | Exacerbates gastric lesions[3] | Prevents gastric lesions[3] |
| Ethanol | Exacerbates gastric damage[5] | Prevents gastric damage[2,5] |
| NSAIDs | Exacerbates gastric lesions[19] | Reduces severity of gastric and intestinal damage[7,8,19,79] |
| Platelet-activating factor | Exacerbates intestinal lesions[56] | Reduces mucosal permeability; reduces severity of intestinal lesions[56] |
| Endothelin-1 | Elevated protein accumulation in lumen of stomach and duodenum[80] | Reduces severity of gastric lesions[80] |
| Ischaemia-reperfusion | Elevated mucosal and vascular permeability[81,82] | Reduced mucosal permeability;[82] reduced gastric lesions;[54] improved survival[83] |
| Stress | Increased ulcer severity[84] and mucosal permeability[85] | Not tested |
| Endotoxin | Increased luminal albumin leakage and mucosal injury[86] | Reduced gastric and intestinal injury[87,88] |

stances is attributable to a complex and dynamic network of factors collectively referred to as 'mucosal defence'. This includes extracellular factors, such as mucus, which helps to prevent abrasive injury to the mucosa and to impair attachment of microbes to the epithelium. The epithelium of the gastrointestinal tract itself forms an important barrier, restricting the movement of large molecules into the mucosa and secreting fluid into the lumen in order to wash away potentially harmful microbes or toxins. The subepithelium is richly innervated by both the extrinsic and enteric nervous systems, which are capable of triggering changes in several of the components of the mucosal defence network when damage to the epithelium is detected or if toxins enter the lamina propria in sufficient quantities. One of the most rapid and important mucosal defence responses is an increase in mucosal blood flow, which acts to dilute the toxic substances, neutralize them and remove them from the tissue before they can accumulate to damaging concentrations. This has been particularly well characterized in the stomach, where back-diffusing acid can trigger sensory afferent neurons beneath the epithelium. The subsequent release of calcitonin gene-related peptide in the vicinity of the submucosal arterioles results in a rapid and profound increase in mucosal blood flow, thereby effectively neutralizing the back-diffusing acid that had triggered the response.

The various components of mucosal defence are co-ordinated and modulated via the release of a number of soluble mediators. For example, prostaglandins play a very important role in the maintenance of mucosal integrity, stimulating virtually every component of the mucosal defence network. Over the past 10 years, substantial data have been generated to suggest that nitric oxide is another key mediator of mucosal defence in the gastrointestinal tract.

### Nitric oxide can protect the GI tract

MacNaughton *et al.*[2] demonstrated that nitric oxide itself or an NO donor could protect the gastric mucosa from damage induced by topically applied ethanol. Moreover, administration of the guanylate cyclase inhibitor, methylene blue, or the nitric oxide scavenger, oxyhaemoglobin, prevented the protective effects of exogenous NO and increased the susceptibility of the gastric mucosa to injury. These findings have now been extended by Kitagawa *et al.*,[3] who showed protective effects of nitric oxide donors in an acid-induced model of damage in the rat, by Lopez-Belmonte *et al.*[4] who demonstrated that nitric oxide donors reduced the severity of ethanol-induced damage, and by Masuda *et al.*[5] who demonstrated that blockade of nitric oxide synthesis greatly increased the susceptibility of the stomach to damage induced by ethanol. A summary of the studies implicating nitric oxide in gastrointestinal mucosal resistance to injury is provided in Table 1.

The ability of nitric oxide to reduce the severity of damage induced by nonsteroidal anti-inflammatory drugs (NSAIDs)[6] has recently been exploited in an attempt to produce NSAIDs which are not ulcerogenic. By attaching a nitric oxide releasing moiety to standard NSAIDs, the ulcerogenic effects of these compounds was greatly reduced without adversely affecting their anti-inflammatory properties.[6–8] These compounds appear to release nitric oxide very slowly and in small amounts. Thus, the compounds do not affect systemic arterial blood pressure, but the release of NO is sufficient to maintain gastric mucosal blood flow and prevent leukocyte adherence to post-capillary venules.[7,8] Decreased mucosal blood flow and leukocyte adherence are observed with standard NSAIDs and are believed to contribute to the pathogenesis of NSAID-induced gastropathy.[9]

There is also recent evidence to suggest that endogenous nitric oxide derived from the inducible isoform of nitric oxide synthase (NOS2) is capable of protecting the stomach, which flies in the face of claims that the large amounts of nitric oxide produced via this enzyme will cause tissue injury. Tepperman and Soper[10] demonstrated that administration of endotoxin to rats led to a significant increase in NOS2 activity within the gastric mucosa. When the stomach was challenged by intragastric administration of ethanol, the rats previously given endotoxin were found to be significantly more resistant to damage. That this protection was mediated via nitric oxide was supported by their observation that administration of L-NAME (a non-selective NOS inhibitor) or dexamethasone inhibited the protective effects associated with endotoxin treatment. In the case of dexamethasone, they demonstrated a selective inhibition of the inducible nitric oxide synthase within the gastric mucosa.

### Nitric oxide modulates mucus secretion

Mucus secretion by the epithelium is believed to form one of the primary levels of mucosal defence against acid in the stomach and duodenum. Mucus also plays a key role in preventing abrasive injury to the gut epithelium, in limiting the access of luminal bacteria to the epithelial surface and as an anti-oxidant.[11] Bicarbonate is produced in small amounts relative to the amount of acid produced by the stomach, but when trapped within mucus on the surface of the epithelium can form a pH gradient. Nitric oxide appears to be one of the most important signals for mucus secretion. Brown *et al.*[12] demonstrated that nitric oxide donors and a cGMP analogue could stimulate mucus release. Furthermore, Price *et al.*[13] reported that nitric oxide was the mediator responsible for cholinergic-stimulated mucus release.

### Nitric oxide mediates mucosal hyperaemic responses

As outlined above, a very important component of mucosal defence is the elevation of mucosal blood flow that occurs when the mucosa is exposed to an irritant. Blockade of this response, by ablation of sensory afferent neurons, renders the mucosa unable to withstand even brief exposure to topical irritants such as 15% ethanol.[14] The reactive hyperaemic response is a nitric oxide-dependent response. Calcitonin gene-related peptide accounts for the largest component of the gastric reactive hyperaemic response,[15] and its effects on vascular smooth muscle tone are mediated through the generation, presumably by the vascular endothelium, of nitric oxide.[16] Prior treatment of rats with inhibitors of nitric oxide synthase abolishes the reactive hyperaemic response to topical irritants and greatly increases the susceptibility of the stomach to damage.

Nitric oxide also plays a key role in modulating basal gastric blood flow, since it can be reduced by administration of NOS inhibitors.[17] Moreover, this reduction of mucosal blood flow by NOS inhibitors probably underlies their ability to increase the susceptibility of the gastric mucosa to injury induced by various topical irritants, such as ethanol and NSAIDs.[4,18,19]

### Nitric oxide is important in repair of mucosal injury

Repair of gastrointestinal injury can be subdivided into two major types. When damage is limited to the most superficial layers of cells, rapid repair of the epithelium is possible through the process of restitution. This involves migration of healthy cells from the uninjured margins, over the exposed basement membrane.[20] The restitution process *in vivo* is dependent upon the maintenance, in the region of injury, of a micro-environment of near neutral pH (the basement membrane is very susceptible to damage by acid and restitution will not occur if the basement membrane is damaged).[21] Mucosal hyperaemia and the leakage of plasma at the site of injury is responsible for maintaining the neutral pH microenvironment. Takeuchi *et al.*[22] recently demonstrated that blockade of nitric oxide production results in an impairment of the vascular response and the consequent alkaline flux into the lumen and in turn, to an impairment of the restitution process.

The second major type of repair that occurs in the gastrointestinal tract is that associated with injury that involves the full thickness of the mucosa, and perhaps even the submucosa and muscularis (i.e. true ulcers). This process involves the formation of granulation tissue at the ulcer base, the process of angiogenesis and the subsequent re-establishment of glandular structure (this occurs primarily from the margins of the ulcer). While the mechanisms of action are yet to be determined, there is now clear evidence that nitric oxide can accelerate this healing process. Konturek *et al.*[23] first demonstrated that administration of a nitric oxide donor (glyceryl trinitrate) significantly accelerated the healing of gastric ulcers in the rat. They also showed that administration of an inhibitor of nitric oxide synthesis impaired ulcer healing. These results were recently confirmed by Elliott *et al.*[24] who showed that oral but not intraperitoneal glyceryl trinitrate accelerated ulcer healing in the rat. Moreover, administration of a nitric oxide-releasing NSAID derivative, but not the parent NSAID, significantly accelerated ulcer healing.[24]

### Nitric oxide regulates intestinal mucosal barrier function

Inhibition of nitric oxide synthase results, within minutes, in a substantial increase in small intestinal epithelial permeability. For example, the transepithelial movement of a marker such as $^{51}$Cr-EDTA is greatly enhanced following administration of the NO synthase inhibitor L-NAME.[25] This increase in epithelial permeability can also be induced by other inhibitors of nitric oxide synthesis, and can be reversed by nitric oxide donors, L-arginine or cGMP analogues. On the other hand, administration of biologically inactive enantiomers of the NO synthase inhibitors had no effect on epithelial permeability. This role for nitric oxide in the intestine has now been demonstrated in the cat,[25] guinea-pig[26] and rat.[27]

It is important to note that in the studies described above, inhibition of nitric oxide synthesis did not result in widespread disruption of the epithelium. Indeed, the permeability of the epithelium to molecules such as albumin (MW 60 000), was not affected by the inhibitors of NO synthase. Moreover, the effect of NO synthase inhibition on permeability to EDTA was very rapidly reversed by administration of an NO donor, demonstrating that the permeability change was not due to damage of the epithelium or to the tight junctions. As epithelial cells in the gastrointestinal tract have the capacity to produce nitric oxide,[28] it is possible that these cells can exert autoregulation of their own permeability. The factors that increase or decrease nitric oxide production by the epithelium have not yet been well characterized, although it is interesting to note that the reduction of epithelial permeability that can be induced by interferon-gamma was recently shown to be mediated via the production of nitric oxide.[29] Of course, numerous other cells in the lamina propria, including enteric neurons, mast cells and macrophages, also have the capacity to produce nitric oxide, and could therefore modulate epithelial permeability. There is now strong evidence for mast cells exerting such control through the release of nitric oxide (see below).

### Nitric oxide modulates the mucosal immune system

The mucosal immune system plays an essential role in protecting us from the enormous quantities of microbes, microbial products and other antigens present in the lumen of the gut. The normal gut could be said to be in a state of 'controlled inflammation'. While there is a constant exposure of the gut to substances that could stimulate an immune response, which could lead to inappropriate injury to the host tissue, a number of mediators act to keep the inflammatory response in check. Included among these down-regulatory molecules are interleukin-10, prostaglandins and nitric oxide.

The ability of nitric oxide to down-regulate immunocyte function has been demonstrated in a number of cell systems. For example, macrophage-derived NO has been suggested to suppress the proliferation of T lymphocytes and to mediate several aspects of macrophage function.[30] As outlined below, nitric oxide is a potent suppressor of neutrophil function, inhibiting both adherence and activation. With respect to the gastrointestinal tract, perhaps the best characterized immunoregulatory effect of nitric oxide is its ability to inhibit mediator release from mast cells. Salvemini *et al.*[31] first reported the ability of exogenous nitric oxide to inhibit release of histamine from peritoneal mast cells. These studies were later extended by Masini *et al.*,[32] who demonstrated that mast cells could produce nitric oxide, and by Hogaboam *et al.*,[33] who reported that the nitric oxide produced by the mast cell autoregulated the release of other inflammatory mediators, such as platelet-activating factor (PAF). The ability of the mast cell to produce nitric oxide could be greatly enhanced by very brief exposure to interleukin-1.[33] Nitric oxide release from mast cells also contributes to the ability of these cells to kill target tumour cells.[34]

These *in vitro* studies of mast cells have now been extended to *in vivo* models. Kanwar *et al.*[27] demonstrated that administration of a nitric oxide synthase inhibitor resulted in a marked increase in serum levels of a protease specific to mucosal mast cells. Using histochemical methods, degranulation of connective tissue-type mast cells in the mesentery was also demonstrated.[35] Associated with the degranulation of mast cells was a rapid increase in permeability of the intestinal epithelium to the small molecular weight marker, EDTA.[27] The permeability changes observed could be prevented by pre-treating the rats with various mast cell stabilizers or with receptor antagonists to PAF or the histamine H-1 receptor. Taken together, these studies demonstrate that inhibition of nitric oxide synthesis leads to mast cell degranulation. The release of mediators such as histamine and PAF results in increases in intestinal epithelial permeability. In addition, inhibition of nitric oxide synthesis led to increased numbers of leukocytes adhering to mesenteric post-capillary venules.[36] This effect, which is discussed in more detail below, also appeared to be mast cell-dependent.[35]

Similar findings have been reported by Kurose *et al*,[37] who studied the effects of ischaemia–reperfusion on intestinal mast cells. They visually assessed mast cell integrity after 30 min of reperfusion of ischaemic mesentery and noted a significant increase in degranulated mast cells that was entirely inhibited by nitric oxide donors. They also demonstrated that endogenous nitric oxide was reduced by 90% following the period of ischaemia, while supplementation of the tissue with nitric oxide donors significantly reduced leukocyte infiltration and microvascular dysfunction. The authors concluded that the reduction in nitric oxide production associated with ischaemia-reperfusion led to destabilization of mast cells, as a consequence of reduced nitric oxide production, and the subsequent release of pro-adhesive molecules such as histamine, PAF and leukotrienes. It is these mediators which ultimately elicited the inflammatory response.

Although the platelet is generally viewed in the context of its important role in thrombosis and coagulation, there is considerable evidence that platelets also contribute significantly to inflammatory processes.[38] Nitric oxide is a very potent inhibitor of platelet aggregation and adherence,[39] and may therefore exert anti-inflammatory effects by suppressing platelet activation. Nishida *et al.*[40] recently reported that suppression of nitric oxide synthesis during administration of endotoxin profoundly increased platelet and fibrin deposition in liver sinusoids. They suggested that inhibition of endogenous nitric oxide results in platelet aggregation which contributes to plugging of the sinusoids and tissue injury. Harbrecht *et al.*[41] also suggested an important role for inducible NOS in preventing thrombus formation in situations of endotoxaemia. They found that suppression of NO synthesis in endotoxaemic animals led to intrahepatic thrombus formation and oxygen radical-mediated liver damage.

### Nitric oxide inhibits neutrophil adherence and activation

Neutrophils are a major contributor to gastrointestinal injury, including that associated with inflammatory bowel disease[42] and *Helicobacter pylori*-associated ulcer disease.[43,44] Moreover, a key role for neutrophils as effectors of tissue damage has been demonstrated in various experimental models of intestinal injury and inflammation, including that induced by ischaemia–reperfusion,[45,46] PAF,[47] indomethacin[48–50] and ethanol.[51,52] The evidence for a role for neutrophils in these models includes the observations that: (1) neutrophils infiltrate the inflamed tissue; (2) rendering animals neutropenic reduces tissue injury; and (3) antibodies against neutrophil and/or endothelial adhesion molecules reduce the extent of injury.

Nitric oxide has been suggested as an important modulator of neutrophil adherence to the vascular endothelium. Inhibition of nitric oxide synthesis leads to elevated numbers of neutrophils adhering to walls of vessels.[53] As outlined above, this may be in part a mast cell-dependent phenomenon. Based on these observations, it has been suggested that one of the mechanisms through which nitric oxide can reduce injury in the gastrointestinal tract is through inhibition of neutrophil recruitment to the tissue. Indeed, in numerous studies, administration of nitric oxide donors reduced gastrointestinal myeloperoxidase activity (an index of tissue granulocyte numbers) as well as reducing tissue injury. Andrews *et al.*[54] demonstrated that fewer neutrophils infiltrated the post-ischaemic gastric mucosa following nitroprusside or acetylcholine administration and this regimen of nitric oxide generators protected the reperfused tissue. Kurose *et al.*[55] demonstrated that administration of nitric oxide donors resulted in a marked attenuation of leukocyte adhesion and emigration, as well as vascular dysfunction in the post-ischaemic mesentery. Mackendrick *et al.*[56] demonstrated that inhibition of nitric oxide production resulted in an exacerbation of PAF-induced neutrophil influx into the mucosa as well as an increase in tissue injury. As mentioned above, the addition of a nitric oxide-releasing moiety to standard NSAIDs resulted in prevention of the neutrophil adherence to mesenteric post-capillary venules normally seen with the parent NSAID.[7]

It should be noted that prevention of infiltration of circulating neutrophils into the intestine may not fully explain the protective effects of nitric oxide. For example, in experimental ischaemia–reperfusion-induced injury, circulating neutrophils appear to contribute significantly to the microvascular but not the mucosal dysfunction.[57] This is based on the fact that pretreatment with an anti-CD18 antibody, which prevents leukocyte adhesion to post-capillary venules, significantly attenuated the increase in microvascular permeability but did not affect the rise in mucosal permeability.[57] On the other hand, nitric oxide donors have been shown to reduce both microvascular and mucosal injury in the intestine. Since adhering neutrophils do not mediate the mucosal permeability alterations in reperfused intestine, then nitric oxide must have other beneficial effects in addition to its anti-adhesive properties. One possibility is that nitric oxide can inhibit functions of neutrophils other than their ability to adhere to the vascular endothelium. For example, nitric oxide may directly inactivate the enzyme responsible for the oxidative burst that is generated by activated neutrophils. Clancy *et al.*[58] have demonstrated that nitric oxide can inhibit superoxide production from neutrophils by directly inhibiting NADPH oxidase. Therefore, in addition to scavenging superoxide (discussed below), nitric oxide can prevent the synthesis of superoxide and associated oxidants including hydrogen peroxide. Thus, in addition to preventing leukocyte adhesion and infiltration into the reperfused intestine, nitric oxide may also directly affect the cytotoxicity of neutrophils by altering the ability of this cell to produce oxidants. Other inhibitory effects of nitric oxide donors on neutrophil function have also been reported.[59–61]

### Nitric oxide is an anti-oxidant

In addition to being anti-adhesive, nitric oxide has the capacity to inhibit reactive oxygen metabolites, including superoxide anion, and can prevent the cellular damage attributable to hydrogen peroxide.[62,63] Both superoxide and hydrogen peroxide have been implicated in the mucosal injury associated with ischaemia–reperfusion[64] and the administration of ethanol[65–67] or NSAIDs.[68] Nitric oxide may play a role in reducing the injury in these models through its ability to inactivate oxidants. Indeed, nitric oxide has been shown to rapidly react with superoxide to abolish its biological activity *in vitro*.[62] There is also evidence suggesting that this occurs *in vivo*, since the anti-oxidant capacity of plasma was doubled by administration of nitric oxide donors at concentrations that prevented reperfusion-induced mucosal injury.[63] Other *in vivo* experiments demonstrated that administration of a superoxide-generating system could induce at least one feature of inflammation (neutrophil influx) and both superoxide dismutase and NO donors directly inhibited this response, further suggesting a potential anti-oxidant ability of these NO donors.[63] Finally, Wink *et al.*[69] recently proposed a protective role for NO donors in cellular injury induced by hydrogen peroxide. Since catalase, which is a hydrogen peroxide-detoxifying agent, can also reduce intestinal inflammation,[64] the possibility that NO donors counter both superoxide and hydrogen peroxide-induced tissue injury cannot be excluded.

### Large amounts of nitric oxide do not cause intestinal injury

It has repeatedly been suggested that production of 'large' amounts of nitric oxide, such as may occur when the inducible form of NOS is present, will result in damage within the gastrointestinal tract or, indeed, in other tissues. In an attempt to test this hypothesis, *in vivo* and *in vitro* studies on the feline intestine and human epithelial cells, respectively, were performed which involved local infusion of high concentrations of nitric oxide donors (CAS 754 or SIN-1) into autoperfused segments of cat ileum.[70] Rather than looking for overt tissue damage, very subtle functional alterations were used as indices of intestinal dysfunction. These included measurements of both microvascular and mucosal permeability. These studies clearly demonstrated that local infusion of compounds that would release 'large' amounts of NO failed to cause changes in microvascular permeability, changes in the integrity of the mucosal barrier or impairment in the functional aspects (absorption or secretion) of the small bowel. Furthermore, when these NO donors were added to endothelial or epithelial cells *in vitro*, they failed to produce detectable damage. This is in sharp contrast to the profound injury (i.e. lysis) observed when a hypoxanthine/xanthine oxidase system, which generates superoxide anions, was added to the cultured cells. Similar observations have been reported by others. For example, nitric oxide released from DEA/NO or spermine–NO themselves did not cause cytotoxicity and in fact prevented cellular injury to Chinese hamster lung fibroblasts or rat mesencephalic dopaminergic cells induced by either hydrogen peroxide or superoxide.[69] These data raise some questions about the contention that nitric oxide *per se* directly injures cells of the intestine or other organs.

It might be argued that large amounts of nitric oxide may only produce tissue injury if released in a setting of active inflammation. In an attempt to test this hypothesis, further experiments using an autoperfused segment of cat intestine were performed, but in addition to administering nitric oxide donors, the pro-inflammatory mediator PAF was infused.[70] By itself, PAF caused a profound increase in both mucosal and microvascular permeability. Concomitant administration of the nitric oxide donor, CAS 754, did not augment these permeability changes; in fact, the nitric oxide donor *inhibited* the permeability enhancing effects of PAF.

### What about peroxynitrite?

Nitric oxide is often described as being highly reactive and a gas. In biological settings, however, it is neither.[71] Nitric oxide reacts very slowly with most biological molecules and therefore its proposed cytotoxicity is dependent upon conversion to much more reactive oxidants. In fact, much of the apparent cytotoxicity of nitric oxide has been attributed to its reaction with superoxide anion to form peroxynitrite.[72] Peroxynitrite is itself cytotoxic, and it can rapidly decompose to the highly reactive and toxic hydroxyl radical (OH•) and nitrogen dioxide ($NO_2$•).[71] The tissue damage that can be caused by peroxynitrite is attributable to its ability to oxidize sulfhydryl groups within cells, thereby depleting an important scavenging mechanism. This leads to increased susceptibility of the cell to oxidant-induced damage to membrane lipids and proteins, various enzymes and DNA.[73]

There are a couple of points that should be considered with respect to the putative role of peroxynitrite in intestinal injury. Rubbo *et al.*[74] recently reported that NO actually inhibits peroxynitrite-induced lipid peroxidation. While NO could participate in the generation of peroxynitrite, by reacting with superoxide anion, and had the capacity to cause lipid peroxidation, this only occurred when the concentrations of NO and superoxide anion present were equal. When NO concentrations were increased, as may well be the case in situations of inflammation, NO inhibited peroxynitrite-dependent lipid peroxidation.

What evidence is there supporting a role for peroxynitrite in the production of gastrointestinal injury? A problem with attempting to provide such evidence is the lack of a selective inhibitor of the formation or actions of this substance. It is possible to stain tissues for the presence of nitrotyrosine, a product produced when peroxynitrite reacts with tyrosine residues in a tissue.[71] Miller *et al.*[26] demonstrated positive staining for nitrotyrosine in inflamed ileum of guinea-pig. The fact that inhibition of nitric oxide synthase resulted in diminished staining for these products supports the concept that the nitrotyrosine was derived from a reaction dependent on nitric oxide synthesis.[26] This evidence supports the concept that peroxynitrite is formed under conditions of intestinal inflammation, but does not address whether or not it contributes to the tissue injury. Blockade of nitric oxide synthase with L-NAME has been shown to reduce the severity of experimental colitis[75] and experimental ileitis.[26,76] Administration of aminoguanidine, which is a more selective inhibitor for inducible NOS, produced an even more impressive reduction of injury and inflammation.[77] While these data support a role for nitric oxide in producing tissue injury and inflammation, they do not directly address the role of peroxynitrite. There is also evidence that intracolonic administration of peroxynitrite will cause extensive damage and inflammation in the rat.[78] Again, however, this does not address the issue of whether or not endogenous peroxynitrite production contributes to tissue injury in colitis. It is clear that real progress in answering this question will be dependent upon the development of highly selective inhibitors of the inducible isoform of NOS, and improved methods for detecting the production of peroxynitrite *in vivo*.

Finally, some caution should be exercised when interpreting the results from studies in which L-NAME is chronically administered and found to reduce the severity of experimental intestinal inflammation. While it is possible that this effect of L-NAME is attributable to suppression of nitric oxide synthesis, and indicative of NO playing a role in the production of tissue injury, there are other possible explanations. First, L-NAME is capable of reducing blood flow to tissues, and in doing so, may reduce the influx of granulocytes. Secondly, L-NAME may cause leukocyte adhesion in tissues other than the one being studied, therefore resulting in an apparent decrease in infiltration of these inflammatory cells into the intestine. Thirdly, in light of the observed ability of L-NAME to cause degranulation of mast cells in the gastrointestinal tract,[27] one must consider the possibility that with chronic administration of this compound there could be a progressive depletion of inflammatory mediators from these cells. It is therefore possible that such a depletion could contribute to the beneficial effects of L-NAME administration over a prolonged period of time. Finally, virtually all the experimental models of intestinal inflammation are dependent upon the presence within the gut lumen of microbes. It is entirely possible that chronic L-NAME administration alters the nature and/or numbers of microbes within the gut lumen and in doing so, alters the magnitude of the mucosal inflammatory response. These alternative explanations for the beneficial effects of chronic L-NAME administration in experimental models of gastrointestinal inflammation have yet to be systematically evaluated.

### Conclusions

There can be little doubt that nitric oxide is among the most important mediators of gastrointestinal mucosal defence, influencing virtually every component of the mucosal defence network. There is emerging evidence that NO is an important endogenous scavenger of various free radical species, and that suppression of NO synthesis can lead to oxidant-mediated tissue injury. More controversial is the putative role of NO, when produced in 'large' amounts via the inducible isoform of NOS, in the production of gastrointestinal injury. While there is evidence that chronic suppression of NO synthesis can reduce some indices of tissue injury and inflammation in experimental models, these effects may not be due to inhibition of the production of cytotoxic concentrations of NO. Indeed, there is convincing evidence that administration of large amounts of NO does not cause detectable damage to the mucosa or vasculature of the intestine. There is also recent *in vitro* data which raise questions about the role of peroxynitrite in the production of tissue injury; specifically, whether conditions ever exist, physiologically or pathophysiologically, in which this substance would be produced in sufficient quantities to cause significant injury. In our view, the vast majority of available data point to nitric oxide serving a critical role in protecting the gastrointestinal mucosa from injury induced by reactive oxygen metabolites and other cytotoxic substances.

### References

1. Furchgott RF, Zawadzki JV. The obligatory role of endothelial cells in the relaxation of arterial smooth muscle by acetylcholine. *Nature* 1980; **288:** 373–376.
2. MacNaughton WK, Cirino G, Wallace JL. Endothelium-derived relaxing factor (nitric oxide) has protective actions in the stomach. *Life Sci* 1989; **45:** 1869–1876.
3. Kitagawa H, Takeda F, Kohei H. Effect of endothelium-derived relaxing factor on the gastric lesion induced by HCl in rats. *J Pharm Exp Ther* 1990; **253:** 1133–1137.
4. Lopez-Belmonte J, Whittle BJR, Moncada S. The actions of nitric oxide donors in the prevention or induction of injury to the rat gastric mucosa. *Br J Pharmacol* 1993; **108:** 73–78.
5. Masuda E, Kawani S, Nagano K, *et al.* Endogenous nitric oxide modulates ethanol-induced gastric mucosal injury in rats. *Gastroenterology* 1995; **108:** 58–64.
6. Wallace JL, Reuter BK, Cirino G. Nitric oxide-releasing non-steroidal anti-inflammatory drugs: a novel approach for reducing gastrointestinal toxicity. *J Gastroenterol Hepatol* 1994; **9:** S40–S44.
7. Wallace JL, Reuter B, Cicala C, McKnight W, Grisham MB, Cirino G. Novel nonsteroidal anti-inflammatory drug derivatives with markedly reduced ulcerogenic properties in the rat. *Gastroenterology* 1994; **107:** 173–179.
8. Wallace JL, Reuter B, Cicala C, McKnight W, Grisham M, Cirino G. A diclofenac derivative without ulcerogenic properties. *Eur J Pharmacol* 1994; **257:** 249–255.
9. Wallace JL. Gastric ulceration: critical events at the neutrophil-endothelium interface. *Can J Physiol Pharmacol* 1993; **71:** 98–102.
10. Tepperman BL, Soper BD. Nitric oxide synthase induction and cytoprotection of rat gastric mucosa from injury by ethanol. *Can J Physiol Pharmacol* 1994; **72:** 1308–1312.
11. Grisham MB, Von Ritter C, Smith BF, Lamont JT, Granger DN. Interaction between oxygen radicals and gastric mucin. *Am J Physiol* 1987; **253:** G93–G96.
12. Brown JF, Keates AC, Hanson PJ, Whittle BJR. Nitric oxide generators and cGMP stimulate mucus secretion by rat gastric mucosal cells. *Am J Physiol* 1993; **265:** G418–G422.
13. Price KJ, Hanson PJ, Whittle BJR. Stimulation by carbachol of mucus gel thickness in rat stomach involves nitric oxide. *Eur J Pharmacol* 1994; **263:** 199–202.
14. Holzer P, Livingston EH, Saria A, Guth PH. Sensory neurons mediate protective vasodilatation in rat gastric mucosa. *Am J Physiol* 1991; **260:** G363–G370.
15. Li D-S, Raybould HE, Quintero E, Guth PH. Role of calcitonin gene-related peptide in gastric hyperemic response to intragastric capsaicin. *Am J Physiol* 1991; **261:** G657–G661.
16. Lippe IT, Holzer P. Participation of endothelium-derived nitric oxide but not prostacyclin in the gastric mucosal hyperaemia due to acid back-diffusion. *Br J Pharmacol* 1992; **105:** 708–714.
17. Piqué JM, Whittle BJR, Esplugues JV. The vasodilator role of endogenous nitric oxide in the rat gastric microcirculation. *Eur J Pharmacol* 1989; **174:** 293–296.
18. Assreuy J, Cunha FQ, Liew FY, Moncada S. Feedback inhibition of nitric oxide synthase activity by nitric oxide. *Br J Pharmacol* 1993; **108:** 833–837.
19. Whittle BJR, Lopez-Belmonte J, Moncada S. Regulation of gastric mucosal integrity by endogenous nitric oxide: interactions with prostanoids and sensory neuropeptides in the rat. *Br J Pharmacol* 1990; **99:** 607–611.
20. Silen W, Ito S. Mechanisms for rapid re-epithelialization of the gastric mucosal surface. *Ann Rev Physiol* 1985; **47:** 217–229.
21. Wallace JL, McKnight GW. The mucoid cap over superficial gastric damage in the rat. A high-pH microenvironment dissipated by nonsteroidal anti-inflammatory drugs and endothelin. *Gastroenterology* 1990; **99:** 295–304.
22. Takeuchi K, Ohuchi T, Okabe S. Endogenous nitric oxide in gastric alkaline response in the rat stomach after damage. *Gastroenterology* 1994; **106:** 367–374.
23. Konturek SJ, Brzozowski T, Majka J, Pytko-Polonczyk J, Stachura J. Inhibition of nitric oxide synthase delays healing of chronic gastric ulcers. *Eur J Pharmacol* 1993; **239:** 215–217.
24. Elliott SN, McKnight W, Cirino G, Wallace JL. A nitric oxide-releasing nonsteroidal anti-inflammatory drug accelerates gastric ulcer healing in rats. *Gastroenterology* 1995; **109:** 524–530.
25. Kubes P. Nitric oxide modulates epithelial permeability in the feline small intestine. *Am J Physiol* 1992; **262:** G1138–G1142.
26. Miller MJS, Sadowska-Krowicka H, Aruemol SC, Kakkis JL, Clark DA. Amelioration of chronic ileitis by nitric oxide synthase inhibition. *J Pharm Exp Ther* 1993; **264:** 11–16.
27. Kanwar S, Wallace JL, Befus D, Kubes P. Nitric oxide synthesis inhibition increases epithelial permeability via mast cells. *Am J Physiol* 1994; **266:** G222–G229.
28. Brown JF, Tepperman BL, Hanson PJ, Whittle BJR, Moncada S. Differential distribution of nitric oxide synthase between cell fractions isolated from the rat gastric mucosa. *Biochem Biophys Res Commun* 1992; **184:** 680–685.
29. Unno N, Menconi MJ, Smith M, Fink MP. Nitric oxide mediates interferon-gamma-induced hyperpermeability in cultured human intestinal epithelial monolayers. *Crit Care Med* 1995; **23:** 1170–1176.
30. Albina JE, Reichner JS. Nitric oxide in inflammation and immunity. *New Horizons* 1995; **3:** 46–64.
31. Salvemine D, Masini E, Anggard E, Mannaioni PF, Vane J. Synthesis of nitric oxide-like factor from L-arginine by rat serosal mast cells: stimulation of guanylate cyclase and inhibition of platelet aggregation. *Biochem Biophys Res Commun* 1990; **169:** 596–601.
32. Masini E, Salvemini D, Pistelli A, Mannaioni PF, Vane JR. Rat mast cells synthesize a nitric oxide like-factor which modulates the release of histamine. *Agents Actions* 1991; **33:** 61–63.
33. Hogaboam CM, Befus AD, Wallace JL. Modulation of rat mast cell reactivity by IL-1 beta. Divergent effects on nitric oxide and platelet-activating factor release. *J Immunol* 1993; **151:** 3767–3774.
34. Bissonnette EY, Hogaboam CM, Wallace JL, Befus AD. Potentiation of tumor necrosis factor-α-mediated cytotoxicity of mast cells by their production of nitric oxide. *J Immunol* 1991; **147:** 3060–3065.
35. Kubes P, Kanwar S, Niu X-F, Gaboury J. Nitric oxide synthesis inhibition induces leukocyte adhesion via superoxide and mast cells. *FASEB J* 1993; **7:** 1293–1299.
36. Kubes P, Granger DN. Nitric oxide modulates microvascular permeability. *Am J Physiol* 1992; **262:** H611–H615.
37. Kurose I, Anderson DC, Miyasaka M, *et al.* Molecular determinants of reperfusion-induced leukocyte adhesion and vascular protein leakage. *Circ Res* 1994; **74:** 336–343.
38. Kazura JW. Platelet-neutrophil interaction: modulation of the inflammatory response. *J Lab Clin Med* 1989; **114:** 469–470.
39. Radomski MW, Vallance P, Whitley G, Foxwell N, Moncada S. Platelet adhesion to human vascular endothelium is modulated by constitutive and cytokine induced nitric oxide. *Cardiovasc Res* 1993; **27:** 1380–1382.
40. Nishida J, McCuskey RS, McDonnell D, Fox ES. Protective role of nitric oxide in hepatic microcirculatory dysfunction during endotoxemia. *Am J Physiol* 1994; **267:** G1135–G1141.
41. Harbrecht BG, Billiar TR, Stadler J, *et al.* Inhibition of nitric oxide synthesis during endotoxemia promotes intrahepatic thrombosis and an oxygen radical-mediated hepatic injury. *J Leuk Biol* 1992; **52:** 390–394.
42. Hermanowicz A, Gibson PR, Jewell DP. The role of phagocytes in inflammatory bowel disease. *Clin Sci* 1985; **69:** 241–249.
43. Craig PM, Territo MC, Karnes WE, Walsh JH. *Helicobacter pylori* secretes a chemotactic factor for monocytes and neutrophils. *Gut* 1992; **33:** 1020–1023.
44. Rautelin H, Blomberg B, Fredlund H, Järnerot G, Danielsson D. Incidence of *Helicobacter pylori* strains activating neutrophils in patients with peptic ulcer disease. *Gut* 1993; **34:** 599–603.
45. Granger DN. Role of xanthine oxidase and granulocytes in ischemia–reperfusion injury. *Am J Physiol* 1988; **255:** H1269–H1275.
46. Hernandez LA, Grisham MB, Twohig B, Arfors KE, Harlan JM, Granger DN. Role of neutrophils in ischemia–reperfusion-induced microvascular injury. *Am J Physiol* 1987; **253:** H699–H703.
47. Kubes P, Suzuki M, Granger DN. Platelet-activating factor-induced microvascular dysfunction: the role of adherent leukocytes. *Am J Physiol* 1990; **258:** G158–G163.
48. Wallace JL, Keenan CM, Granger DN. Gastric ulceration induced by nonsteroidal anti-inflammatory drugs is a neutrophil-dependent process. *Am J Physiol* 1990; **259:** G462–G467.
49. Wallace JL, Arfors KE, McKnight GW. A monoclonal antibody against the CD18 leukocyte adhesion molecule prevents indomethacin-induced gastric damage in the rabbit. *Gastroenterology* 1991; **100:** 878–883.
50. Alican IT, Coskun A, Corak, Yegen BC, Oktay S, Kurtel H. Role of neutrophils in indomethacin-induced gastric mucosal lesions in rats. *Inflamm Res* 1995; **44:** 164–168.
51. Kvietys PR, Perry MA, Gaginella TS, Granger DN. Ethanol enhances leukocyte-endothelial cell interactions in mesenteric venules. *Am J Physiol* 1990; **259:** G578–G583.
52. Kvietys PR, Twohig B, Danzell J, Specian RD. Ethanol-induced injury to the rat gastric mucosa. Role of neutrophils and xanthine oxidase-derived radicals. *Gastroenterology* 1990; **98:** 909–920.
53. Kubes P, Suzuki M, Granger DN. Nitric oxide: an endogenous modulator of leukocyte adhesion. *Proc Natl Acad Sci USA* 1991; **88:** 4651–4655.
54. Andrews FJ, Malcontenti-Wilson C, O'Brien PE. Protection against gastric ischemia–reperfusion injury by nitric oxide generators. *Dig Dis Sci* 1994; **39:** 366–373.
55. Kurose I, Kubes P, Wolf R, *et al.* Inhibition of nitric oxide production: mechanisms of vascular albumin leakage. *Circ Res* 1993; **73:** 164–171.
56. Mackendrick W, Caplan M, Hsueh W. Endogenous nitric oxide protects against platelet-activating factor-induced bowel injury in the rat. *Pediatr Res* 1993; **34:** 222–228.
57. Kubes P, Hunter JA, Granger DN. Ischemia/reperfusion-induced feline intestinal dysfunction: importance of granulocyte recruitment. *Gastroenterology* 1992; **103:** 807–812.
58. Clancy RM, Leszczynska-Piziak J, Abramson SB. Nitric oxide, and endothelial cell relaxation factor, inhibits neutrophil superoxide production via a direct action on the NADPH oxidase. *J Clin Invest* 1992; **90:** 1116–1121.
59. Siminiak T, Zozulinska D, Wysocki H. Inhibition of polymorphonuclear neutrophil function by nitric oxide donor SIN-1 *in vitro*: relationship to the presence of platelets. *Pharmacol Comm* 1992; **2:** 217–224.
60. McCall TB, Boughton-Smith NK, Palmer RMJ, Whittle BJR, Moncada S. Synthesis of nitric oxide from L-arginine by neutrophils. Release and interaction with superoxide anion. *Biochem J* 1989; **261:** 293–296.
61. McCall T, Whittle BJR, Boughton-Smith NK, Moncada S. Inhibition of FMLP-induced aggregation of rabbit neutrophils by nitric oxide. *Br J Pharmacol* 1988; **95:** 517P.
62. Rubanyi GM, Ho EH, Cantor EH, Lumma WC, Botelho LHP. Cytoprotective function of nitric oxide: inactivation of superoxide radicals produced by human leukocytes. *Biochem Biophys Res Commun* 1991; **181:** 1392–1397.
63. Gaboury J, Woodman RC, Granger DN, Reinhardt P, Kubes P. Nitric oxide prevents leukocyte adherence: role of superoxide. *Am J Physiol.* 1993; **265:** H862–H867.
64. Granger DN, Hollwarth ME, Parks DA. Ischemia–reperfusion injury: role of oxygen-derived free radicals. *Acta Physiol Scand. [Suppl]* 1986; **548:** 47–63.
65. Evangelista AS, Meli A. Influence of antioxidants and radical scavengers on ethanol-induced gastric ulcers in the rat. *Gen Pharm* 1985; **16:** 285–286.
66. Pihan G, Regillo C, Szabo S. Free radicals and lipid peroxidation in the ethanol- or aspirin-induced gastric mucosal injury. *Dig Dis Sci* 1987; **32:** 1395–1401.
67. Terano A, Hiraishi H, Ota S, Shiga J, Sugimoto T. Role of oxygen derived free radicals in ethanol-induced damage in rat stomach. *Gastroenterology* 1986; **90:** 1661.
68. Vaananen PM, Meddings JB, Wallace JL. Role of oxygen-derived free radicals in indomethacin-induced gastric injury. *Am J Physiol* 1991; **261:** G470–G475.
69. Wink DA, Hanbauer IH, Krishna MC, DeGraff W, Gamson J, Mitchell JB. Nitric oxide protects against cellular damage and cytotoxicity from reactive oxygen species. *Proc Natl Acad Sci USA* 1993; **90:** 9813–9817.
70. Kubes P, Reinhardt P, Payne D, Woodman RC. Excess nitric oxide does not cause cellular, vascular or mucosal dysfunction in the cat small intestine. *Am J Physiol* 1995; **269:** G34–G41.
71. Beckman JS. Biochemistry of nitric oxide and peroxynitrite. In: Kubes P. *Nitric Oxide: A Modulator of Cell–Cell Interactions in the Microcirculation.* Austin: R.G. Landes Company, 1995; (in press).
72. Beckman JS, Beckman TW, Chen J, Marshall PA, Freeman BA. Apparent hydroxyl radical production by peroxynitrite: implications for endothelial injury from nitric oxide and superoxide. *Proc Natl Acad Sci USA* 1990; **87:** 1620–1624.
73. Radi R, Beckman JS, Bush KM, Freeman BA. Peroxynitric oxidation of sulfhydryls. The cytotoxic potential of superoxide and nitric oxide. *J Biol Chem* 1991; **266:** 4244–4250.
74. Rubbo H, Radi R, Trujillo M, *et al.* Nitric oxide regulation of superoxide and peroxynitrite-dependent lipid peroxidation. Formation of novel nitrogen-containing oxidized lipid derivatives. *J Biol Chem* 1994; **269:** 26066–26075.
75. Hogaboam CM, Jacobson K, Collins SM, Blennerhassett MG. The selective beneficial effects of nitric oxide inhibition in experimental colitis. *Am J Physiol* 1995; **268:** G673–G684.
76. Seago ND, Thompson JH, Zhang X-J, *et al.* Inducible nitric oxide synthase and guinea-pig ileitis induced by adjuvant. *Med Inflammation* 1995; **4:** 19–24.
77. Thompson JA, Sadowska-Krowicka AH, Rossi J, Clark DA, Miller MJS. Inducible nitric oxide synthase gene expression in guinea pig ileitis: a model of IBD prevented by aminoguanidine. *Gastroenterology* 1994; **106:** A782.
78. Rachmilewitz D, Stamler JS, Karmeli F, *et al.* Peroxynitrite-induced rat colitis—a new model of colonic inflammation. *Gastroenterology* 1993; **105:** 1681–1688.
79. Reuter BK, Cirino G, Wallace JL. Markedly reduced intestinal toxicity of a diclofenac derivative. *Life Sci* 1994; **55:** PL1–PL8.
80. Filep JG, Foldes-Filep E, Rousseau A, Sirois P, Fournier A. Vascular responses to endothelin-1 following inhibition of nitric oxide synthesis in the conscious rat. *Br J Pharmacol* 1993; **110:** 1213–1221.
81. Kubes P. Ischemia/reperfusion in the feline small intestine: a role for nitric oxide. *Am J Physiol* 1993; **264:** G143–G149.
82. Payne D, Kubes P. Nitric oxide donors reduce the rise in reperfusion-induced intestinal mucosal permeability. *Am J Physiol* 1993; **265:** G189–G195.
83. Aoki N, Johnson G, Lefer AM. Beneficial effects of two forms of NO administration in feline splanchnic artery occlusion shock. *Am J Physiol* 1990; **258:** G275–G281.
84. Ogle CW, Qiu BS. Nitric oxide inhibition intensifies cold-restraint induced gastric ulcers in rats. *Experientia* 1993; **49:** 304–307.
85. Coskun T, Yegen BC, Alican I, Peker O, Kurtel H. Cold-restraint stress induced gastric mucosal dysfunction: role of nitric oxide. *Dig Dis Sci* 1995; (in press).
86. Hutcheson IR, Whittle BJR, Boughton-Smith NK. Role of nitric oxide in maintaining vascular integrity in endotoxin-induced acute intestinal damage in the rat. *Br J Pharmacol* 1990; **101:** 815–820.
87. Boughton-Smith NK, Hutcheson IR, Deakin AM, Whittle BJR, Moncada S. Protective effect of *S*-nitroso-*N*-acetyl-penicillamine in endotoxin-induced acute intestinal damage in the rat. *Eur J Pharmacol* 1990; **191:** 485–488.
88. Wallace JL, Cirino G, McKnight GW, Elliott SN. Reduction of gastrointestinal injury in acute endotoxic shock by flurbiprofen nitroxybutylester. *Eur J Pharmacol* 1995; **280:** 63–68.

ACKNOWLEDGEMENTS. The authors are supported by grants from the Medical Research Council of Canada (MRC), the Crohn's and Colitis Foundation of Canada and the Alberta Heart and Stroke Foundation. Dr Wallace is an MRC Scientist and an Alberta Heritage Foundation for Medical Research (AHFMR) Scientist. Dr Kubes is an MRC Scholar and an AHFMR Scholar.

**Received 11 September 1995; accepted 13 September 1995**
